# STRESS AND PSYCHOLOGICAL CHARACTERISTICS PREDICT CLUSTERED HEALTH TRAJECTORIES IN OLDER ADULTS

**DOI:** 10.1093/geroni/igz038.1005

**Published:** 2019-11-08

**Authors:** Myriam V Thoma, Jan Höltge, Shauna L Mc Gee, Andreas Maercker, Mareike Augsburger

**Affiliations:** University of Zürich, Zürich, Switzerland

## Abstract

The first aim of this study was the empirical identification of clustered health trajectories in older individuals, including an expected, more favorable or successful aging (SA) trajectory. The second aim was the identification and analysis of determinants useful for subgroup membership prediction. Particular focus was on early-life and chronic stress, as well as on a broad set of psychological characteristics, such as resilience, personality traits and general affect. A longitudinal survey study with two assessments one year apart has been conducted with older adults (N=224; mean age = 68 years; 72% women). The clustered health trajectories were identified using a longitudinal variant of k-means. For the prediction of subgroup allocation, random forests with conditional interferences were used. The applied machine learning-based approach revealed two latent clustered health trajectories: a ‘constant high health’ (66% of the sample) and a ‘maintaining low health’ trajectory (34%). Chronic stress and positive affect were found to be the most important predictors. Further predictors and their interactions were found to be important for predicting subgroup belonging, including resilience, self-esteem, social support, optimism, as well as negative affect and pessimism. Also, childhood adversities have to be included to predict subgroup belonging. With this study, we were able to show that individuals can be empirically allocated to two separate joint health trajectories in later life over the observation period of one year. In order to understand the current heterogeneity in health in older age, previous and current stress exposure and psychological characteristics have to be taken into account.

